# Transfer of Antioxidant Capacity Through Placenta and Colostrum: β-Carotene and Superoxide Dismutase Collaboratively Enhance Integrated Breeding of Sows and Piglets

**DOI:** 10.3390/antiox14030359

**Published:** 2025-03-18

**Authors:** Jun Huang, Shengkai Li, Jung Yeol Sung, Shiyan Qiao, Xiangfang Zeng, Junyan Zhou

**Affiliations:** 1 College of Animal Science and Technology, Beijing University of Agriculture, Beijing 102206, China; huangjun112@stu.scau.edu.cn (J.H.); lishengkai@bua.edu.cn (S.L.); 2 College of Animal Science and Technology, China Agricultural University, Beijing 100193, China; qiaoshiyan@cau.edu.cn (S.Q.); zengxf@cau.edu.cn (X.Z.); 3 Department of Animal Science, North Carolina State University, Raleigh, NC 27695, USA; jysung@ncsu.edu

**Keywords:** antioxidant capacity, placenta, colostrum, sows, piglets

## Abstract

Sows and piglets face heightened oxidative stress during gestation and lactation, yet strategies to simultaneously mitigate these challenges remain underexplored. This study investigated the effects of β-carotene and superoxide dismutase (SOD) supplementation on 140 Landrace × Yorkshire sows (parity 3–5) randomly assigned to (1) a control; (2) long-term low-dose treatment (25 mg/kg β-carotene, 4 mg/kg SOD, or both) throughout gestation–lactation; or (3) short-term high-dose treatment (100 mg/kg β-carotene, 14 mg/kg SOD, or both) administered 7 days pre/post-weaning and farrowing. Our data indicate that the antioxidants enhanced the productive performance of both sows and piglets, with the most pronounced effect observed in the long-term, low-dose combined administration of β-carotene and SOD. The composite antioxidants significantly improved the systemic antioxidant capacity in sows, while concurrently reducing the cortisol and lipopolysaccharide concentrations in the serum. This enhancement contributed to elevations in serum progesterone and prolactin levels at day 40 of gestation and farrowing, respectively, ultimately increasing the number of weaned piglets and decreasing the backfat loss. In addition, the compound antioxidants improved the serum antioxidant indices of piglets, increased the growth hormone concentrations, and improved the litter weight gain. Mechanistically, the placental upregulation of *CAT*, *GPX1*, and *GLUT3*, alongside Claudin1, Occludin, and ZO-1 expression, underpinned improved nutrient transport and barrier function. These findings demonstrate that β-carotene and SOD synergistically transfer antioxidant capacity via placental and colostrum pathways, offering a viable strategy for integrated sow–piglet management.

## 1. Introduction

Gestation causes anatomical, physiological, and metabolic changes within the maternal body of a sow. These changes are necessary to meet the intrauterine requirements for nutrients and oxygen for the fetus [1]. However, the gestation period is characterized by systemic oxidative stress and chronic inflammation, a condition that is particularly pronounced in hyperprolific sows [2]. This condition is driven by reactive oxygen species (ROS), which are derived from the extensive metabolic demands of both the sow and its fetus [3]. Various factors, including environmental stressors, emotional stress, and related diseases, can disrupt the normal redox status during gestation [4,5]. When the antioxidant system in sows does not effectively scavenge free radicals, the negative impact of oxidative stress is more pronounced. Oxidative stress damages the tissues and cells of the body, which decreases the nutrient transport capacity of the placenta and the reproductive performance of sows. Furthermore, redox imbalances in sows during gestation can potentially induce oxidative stress in the fetus as well, with potential long-lasting consequences for postnatal development [6].

The placenta is crucial in transferring antioxidants from mother to fetus during gestation, which reduces the oxidative stress of the fetus [7,8]. Previous studies have consistently reported that placental and maternal redox dysfunction is highly related to reduced reproductive performance [9,10]. Notably, when the balance of the placenta’s redox system is disrupted, multiple aspects of placental development and function are disrupted, including vascularization, structural integrity, and critical maternal–fetal exchange processes, creating a harmful intrauterine environment for the fetus [11]. Lipopolysaccharide (LPS), a component of Gram-negative bacteria, can induce an inflammatory cascade upon systemic dissemination via the bloodstream, which potentially affects extraintestinal sites, including the placenta [12]. The detrimental effects of LPS exposure on the placenta are well documented, evidenced by inflammation, compromised barrier function, and impaired nutrient transportation, ultimately precipitating reduced reproductive performance [13,14].

Supplementing functional additives with antioxidants such as vitamins is one of the most widely recognized and effective methods to improve human and animal reproduction [15,16]. Under stress conditions, supplementing animals with appropriate amounts of vitamins can potentially enhance their antioxidant capacity, thereby alleviating their oxidative stress [17]. β-Carotene, a lipid-soluble antioxidant, is incorporated into cellular membranes to neutralize lipid peroxides. As a precursor of vitamin A, it effectively scavenges ROS, enhances glutathione peroxidase (GSH-Px) activity in animals, and protects intracellular DNA and proteins from free radical-induced damage [18]. Superoxide dismutase (SOD), an antioxidant metalloenzyme that is naturally occurring in living organisms, has the potential to boost systemic antioxidant defenses through two primary mechanisms: partial absorption via intestinal endocytosis and the modulation of redox signaling pathways. This enzyme plays a vital role in cellular homeostasis by catalyzing the conversion of superoxide anion radicals into molecular oxygen and hydrogen peroxide, thereby maintaining the critical balance between oxidative stress and antioxidant protection within biological systems [19,20]. However, it remains unclear whether the combination of β-carotene and SOD synergistically reduces oxidative stress in both sows and fetuses through the transfer of these antioxidants. Additionally, the effects of β-carotene and SOD supplementation in terms of the concentration and period are not well understood.

In the present study, we deliberately selected sows with a history of large litter sizes as experimental subjects, as they may experience more pronounced oxidative stress during gestation [2]. Initially, we explored the individual and combined supplementation of β-carotene and SOD on sow reproductive performance over both longer and shorter periods. After verifying the significant regulatory effects of the antioxidants on phenotypic indicators such as the number of weaned piglets, backfat loss, milk protein content, and piglet growth performance, we systematically examined the impacts of these antioxidants on oxidative stress and inflammatory responses in sows. Subsequently, we further assessed the antioxidant capacity and hormone secretion status in piglets and sought to uncover the mechanisms underlying the differences observed among the various treatment groups by examining the gene expression in sow placentas.

## 2. Materials and Methods

### 2.1. Animals, Treatments, and Management

All animal experiments were conducted following the guidelines of the China Agricultural University Animal Care and Use Committee (Beijing, China, AW21101202-1-2). This experiment was conducted at the Ningqiang Sano Smart Agriculture Industrial Park Development Co., Ltd. (Ningqiang, China).

One hundred and forty Landrace × Yorkshire sows (parities 3–5) were assigned to seven dietary treatments (one control diet and six supplementary diets), with each sow as a replicate in a completely randomized design, with the weight at weaning of the previous parity as a block (n = 20 per treatment group). Seven sows were removed from the trial before farrowing due to severe lameness or mortality, resulting in 113 sows completing the experiment and being included in the final data analysis (detailed sow production information is provided in Table S1). The first three diets were as follows: CON supplemented with 25 mg/kg β-carotene (L1), 4 mg/kg of SOD (L2), and a combination of both (L3). The other three diets were as follows: CON supplemented with 100 mg/kg β-carotene (S1), 14 mg/kg of SOD (S2), and a combination of both (S3). The L1, L2, and L3 diets were given to respective sows from the end of the previous reproductive cycle until the termination of the trial. In contrast, the S1, S2, and S3 diets were provided 7 days before and after weaning in the previous breeding cycle, as well as 7 days before and after farrowing in the current breeding cycle. Both SOD and β-carotene were sourced from Beijing Hilink Biotechnology Co., Ltd. (Beijing, China). The CON diets for gestation and lactation were formulated to meet their nutrient requirements (NRC, 2012). The ingredients and nutritional levels of the basal diet are shown in Table 1. The calculation of the digestible energy (DE), metabolizable energy (ME), and net energy (NE) content of the ingredients referred to the China National Standard (GB/T 39235-2020) [21]. Chemical analyses of the basal diet in the gestation phase and lactation phase were performed. The crude protein was calculated by multiplying nitrogen by the factor 6.25. Nitrogen was measured according to method 984.13 of the Association of Official Analytical Chemists (AOAC). The content of crude fiber, calcium, phosphorus, and lysine was determined according to the methods of GB/T 6434-2006 [22], GB/T 6436-2018 [23], GB/T 6437-2018 [24], and GB/T 18246-2019 [25], respectively.

Sows were housed in gestation barns (2 × 0.6 m^2^) from the day of weaning until day 109 of gestation, with feeding schedules set at 08:00 and 14:00 h. On day 110 of gestation, the sows were transferred to farrowing barns (2 × 1.5 m^2^) for farrowing preparation. Following farrowing, the sows were fed four times a day, starting with an initial amount of 2 kg and gradually increasing this by 0.5 kg per day until reaching their maximum feed intake. Both gestation and lactation sows were housed in stalls with unrestricted access to water. The gestation barns were maintained at 19 to 23 °C, while the farrowing barns were kept at 23 to 25 °C.

The piglets were managed according to a standard protocol: colostrum intake within 6 h after farrowing and weaning at 25 days. All piglets were evaluated for weight gain and health status before weaning. Each piglet’s health status and diarrhea condition during the experimental period were observed and recorded at 09:00 and 15:00 h every day. The fecal scores of nursery pigs were blindly evaluated according to the following fecal scoring criteria: 0 for normal cylindrical solid feces; 1 for soft but formed feces; 2 for sticky, unformed feces with high moisture content; and 3 for liquid, unformed feces with very high moisture content. Piglets with a fecal score of 2 or 3 for two consecutive days were considered to have diarrhea.

### 2.2. Sample Collection

On day 40 of gestation, the day of farrowing, and the day of weaning, six sows were randomly selected from each treatment group for blood collection via the marginal ear vein. A total of 5 mL of blood was collected, left for 1.5 h, and then centrifuged at 3000 rpm for 15 min to collect serum, which was stored at −20 °C for subsequent analysis. It is important to note that the same six sows were used for each stage. Two days before farrowing, feces samples were collected from six randomly selected sows in each treatment group. On the day of farrowing, 15 mL of colostrum was evenly collected from the anterior, middle, and posterior teats of six randomly selected sows in each treatment group. Furthermore, at the time of farrowing, the umbilical cords of the newborn piglets were tied with a short cotton thread and labeled with a number tag to ensure that each piglet was matched with the corresponding placenta. After the placenta was expelled, approximately 5 g of placental tissue (3 to 5 cm from the umbilical cord insertion point) was collected and rapidly frozen in liquid nitrogen.

Weaning was performed at 25 days post-farrowing based on the piglets’ weight (>5 kg) and health status (absence of diarrhea or respiratory symptoms). On the 14th day of lactation and the day of weaning, 6 piglets were selected from each treatment group and injected with an overdose of sodium pentobarbital. After death, blood was collected from the anterior vena cava. A total of 5 mL of blood was collected from each piglet in an ordinary vacuum blood collection tube. After standing for 1.5 h, the blood was centrifuged at 3000 rpm for 15 min to collect serum, which was divided into 1.5 mL centrifuge tubes and stored at −20 °C for testing.

### 2.3. Chemical Analyses

The content of lactose, milk fat, and milk protein in the colostrum was determined using a fully automated biochemical analyzer (BC-30S) manufactured by Mindray Bio-Medical Electronics Co., Ltd. (Shenzhen, China). The concentrations of IgG (B162520), IgA (B162518), and IgM (B162527) in the colostrum were measured using immunoglobulin assay kits (Hengyuan, Shanghai, China). The SOD (B162441), GSH-Px (B162476), total antioxidant capacity (T-AOC, B162626), nitric oxide synthase (NOS, B162595), H_2_O_2_ (A-029-SH), malondialdehyde (MDA, B162437), TNF-α (HB355-Pg), IL-1β (B162426), IL-6 (HB347-Pg), IL-10 (HB359-Pg), growth hormone (GH, B162555), insulin-like growth factor 1 (IGF-1, HY-50023K), cortisol (HY-50033K), LPS (HB022-Pg), progesterone (HB035-Pg), and prolactin (B162454) were determined using commercial assay kits (Hengyuan, Shanghai, China), according to the manufacturers’ instructions.

### 2.4. Total RNA Extraction and Real-Time Quantitative PCR

According to the manufacturer’s instructions, the total placental RNA was extracted using the RNA extraction kit (Mei5 Biotechnology Co., Ltd., Beijing, China). The quality and concentration of the RNA (A260/A280) were measured using a NanoDrop spectrophotometer. Subsequently, 1 μg of RNA was reverse-transcribed into cDNA using a reverse transcription kit (EZBioscience, Guangzhou, China). A real-time fluorescent quantitative PCR was performed using a 10 μL PCR system comprising 5.0 μL of SYBR Green Premix Ex Taq, 2.6 μL of double-distilled water, 2.0 μL of the cDNA template, and 0.2 μL each of the forward and reverse primers (10 mM), with the primer sequences used in the experiment listed in Table 2 below. The PCR reaction program was as follows: initial denaturation at 95 °C for 1 min; amplification and quantification cycles at 95 °C for 5 s and 58 °C for 34 s, for a total of 35 cycles; a melting curve program at 95 °C for 5 s and 60 °C for 1 min; and a cooling step at 4 °C for 30 s. The PCR reaction CT values were corrected using the housekeeping gene GAPDH, and the relative expression level of the target gene was calculated using the formula 2^−ΔΔCT^.

### 2.5. Western Blotting Analysis

A total of 100 mg of pig placental tissue was employed for the extraction of total protein, facilitated by 0.5 mL of RIPA lysis buffer (Beyotime, Shanghai, China) supplemented with 1% phosphatase inhibitor and 1% PMSF protease inhibitor. Subsequently, the total protein (25 μg per sample) was separated by 10% SDS-PAGE (10% resolving gel) and subsequently transferred to a polyvinylidene difluoride (PVDF) membrane (Millipore, Bedford, MA, USA) using a wet electroblotting system. The membrane was then blocked with 5% skimmed milk powder for 2 h. Following four washes with TBST buffer, the membrane was incubated with β-actin (AB8226, Abcam (Cambridge, UK), 1:5000), ZO-1 (AB96587, Abcam, 1:500), Occludin (AB167161, Abcam, 1:1000), Claudin1 (AB211737, Abcam, 1:2000), HO-1 (66743, Proteintech (Rosemont, IL, USA), 1:1000), Keap1 (AB89901, Abcam, 1:500), P-Nrf2 (BS-2013R, Bioss (Woburn, MA, USA), 1:500), and Nrf2 (BS-1074R, Bioss, 1:100) at 4 °C overnight. After washing, the membrane was incubated with the appropriate HRP goat anti-rabbit (111035-003, Jackson (Lansing, MI, USA), 1:10,000) for 1.5 h at room temperature. Following antibody incubation, the membrane was washed five times with TBST buffer. Chemiluminescent signals were detected using the ECL Plus detection kit (Applygen Technologies Inc., Beijing, China) and visualized on a chemiluminescence imaging analysis system (Tanon, Shanghai, China). Finally, grayscale values were analyzed using image processing software (ImagePro Plus 6.0), and the relative protein expression levels were normalized against β-actin as the internal control.

### 2.6. Bacterial Data Analysis

Genomic DNA was extracted from fresh sow feces utilizing the CTAB method, and its concentration and purity were evaluated via 1% agarose gel electrophoresis. To amplify the V3-V4 variable regions, the primers 341F (5′-CCTAYGGGRBGCASCAG-3′) and 806R (5′-GGACTACNNGGGTATCTAAT-3′) were employed. Following PCR amplification, the products were purified using the Qiagen Gel Purification Kit (Qiagen, Düsseldorf, Germany). Subsequently, sequencing libraries were constructed according to the manufacturer’s guidelines using the TruSeq^®^ DNA PCR-Free Sample Preparation Kit (Illumina, San Diego, CA, USA), incorporating unique index codes for sample identification. The sequencing of 250 bp paired-end reads was performed on an Illumina NovaSeq platform. The raw paired-end reads were merged into single reads for marker generation using the FLASH software (version 34.0.0.175). Data filtering and noise reduction were then conducted with the QIIME software (version 1.91) to obtain a comprehensive list of amplicon sequence variants (ASVs) and their respective features. Subsequently, species annotation was performed on the ASVs to identify the species information associated with each ASV.

### 2.7. Calculation

The litter weight gain was calculated using the following equation:

Litter weight gain (kg) = litter weight at weaning (kg) − litter weight at farrowing (kg).

The diarrhea rate was calculated using the following equation:

Diarrhea rate (%) = number of diarrhea cases in piglets during the experimental period/(total number of piglets per group × experimental days) × 100%.

### 2.8. Statistical Analysis

The PROC MIXED procedure of SAS version 9.4 (SAS Institute, Cary, NC, USA) was used to perform the data analysis. All data were checked for a normal distribution and homogeneous variance using the UNIVARIATE procedure. A sow or nursery pig was considered the experimental unit. Data on the long-term and short-term effects of antioxidants were analyzed separately. Within each period, β-carotene, SOD, and their interaction were included in the model as fixed effects. A random effect of the BW block was included in the model for all measures of reproduction performance. Only the main effects were discussed for responses when the interactions were not significant. The LSMEANS statement was used to calculate the treatment means. The data were analyzed by analysis of variance (ANOVA). Statistical significance was declared at *p* < 0.05, and 0.05 < *p* < 0.10 was considered to indicate a tendency. Tukey’s post hoc test was used to test the differences between groups.

The α-diversity of the fecal bacterial community was assessed by utilizing the Mann–Whitney U test and the Kruskal–Wallis test for statistical analysis. The statistical significance of the principal coordinate analysis (PCoA) for microbial composition comparisons across treatments was ascertained using the QIIME software package (version 1.91), which relied on the Bray–Curtis distance metric for its calculations. Furthermore, linear discriminant analysis effect size (LEfSe) was applied to discern differences at various taxonomic hierarchies, encompassing the phylum, class, order, family, and genus levels.

## 3. Results

### 3.1. Effects of Antioxidants on Reproductive Performance of Sows

To evaluate the effects of long-term and short-term antioxidant supplementation on the reproductive performance of sows, we recorded the litter size, backfat loss, and other indicators of each treatment group. As summarized in Table 3, the number of piglets weaned in the L3 group was significantly higher than that in the CON group (*p* < 0.05). When compared with the CON group, the L3 group exhibited significantly reduced backfat loss (*p* < 0.05). No statistically significant differences were observed in the total number of piglets born, piglets born, and piglets mummified among the groups.

### 3.2. Effects of Antioxidants on the Composition and Immunoglobulin Content of Sow Colostrum

To evaluate the effects of long-term and short-term antioxidant supplementation on the quality of the sow colostrum, we analyzed the colostrum composition and immunoglobulin in each treatment group. As shown in Table 4, compared with the CON group, the milk protein content in the L2, L3, and S1 groups was significantly increased (*p* < 0.05), with the L3 group exhibiting a higher level than the S2 and S3 groups. Regarding the immunoglobulin levels, the IgG concentrations in the L2, L3, S1, S2, and S3 groups were significantly elevated compared with the CON group. Specifically, the IgG levels in the L2 and L3 groups were significantly higher than those in the S2 and S3 groups, while the S1 group showed a significantly higher IgG level than the L1 group. Additionally, the IgM concentrations in the L2 and L3 groups were significantly increased compared with the CON group (*p* < 0.05).

### 3.3. Effects of Antioxidants on Antioxidant Enzyme Activity in Serum, Colostrum, and Placenta

To evaluate whether long-term and short-term antioxidant supplementation could affect the body’s oxidative stress markers, we statistically analyzed the levels of T-AOC, SOD, GSH-Px, NOS, H_2_O_2_, and MDA in the serum, colostrum, and placenta of each treatment group. As indicated in Table 5, in the serum of sows at day 40 of gestation, the SOD content in the L3 and S1 groups was significantly increased compared with the CON group (*p* < 0.05). Additionally, the NOS content in the L1, L2, S1, S2, and S3 groups was significantly elevated compared with the CON group (*p* < 0.05). Moreover, the H_2_O_2_ content in the L2 and S3 groups was significantly reduced compared with the CON group (*p* < 0.05). In the serum of sows at farrowing, the T-AOC content in the L3 group was significantly higher than that in the CON group. Compared with the CON group, the NOS content in the L1, L3, S1, S2, and S3 groups was significantly increased (*p* < 0.05). Additionally, the H_2_O_2_ content in the L2, L3, S1, S2, and S3 groups was significantly reduced compared with the CON group, and the L3 group had a significantly lower H_2_O_2_ level than the other groups. In the serum of sows at weaning, the SOD content in the L3 group was significantly increased compared with the CON group. Additionally, both the L3 and S2 groups showed significantly elevated NOS levels compared with the CON group, with that in the L3 group being higher than in all short-term antioxidant groups.

In the colostrum of sows, the T-AOC content in the S3 group was significantly increased compared with the CON group (*p* < 0.05). Additionally, both the long-term and short-term antioxidant groups showed significantly elevated GSH-Px levels compared with the CON group, with the S1 group exhibiting notably higher GSH-Px content than all long-term antioxidant groups. However, no significant differences were observed in the SOD, NOS, H_2_O_2_, and MDA content among the other treatment groups.

In the placentas of sows, the T-AOC content in the L1, L3, and S2 groups was significantly increased compared with the CON group, with that in the L3 group being higher than in both the S1 and S3 groups (*p* < 0.05). Additionally, the SOD content in the L3 group was significantly elevated compared with the CON group and all short-term antioxidant groups (*p* < 0.05). The NOS content in the L2 and L3 groups was also significantly increased compared with the CON group (*p* < 0.05). Furthermore, the H_2_O_2_ content in all antioxidant groups was significantly reduced compared with the CON group (*p* < 0.05). The MDA content in the L3 group was significantly decreased compared with the CON group (*p* < 0.05).

### 3.4. Effects of Antioxidants on Prolactin and Progesterone Levels in Sow Serum

To evaluate whether long-term versus short-term antioxidant supplementation affected the progesterone levels in sows during pregnancy, we analyzed the serum progesterone and prolactin levels at 40 days of gestation in each treatment group. As indicated in Table 6, at 40 days of gestation, the progesterone levels in the L3, S1, and S3 groups were significantly increased compared with the CON group (*p* < 0.05), with the L3 group showing notably higher progesterone levels than all short-term antioxidant groups. Furthermore, at farrowing, the prolactin levels in the long-term antioxidant groups and the S2 and S3 groups were significantly elevated compared with the CON group (*p* < 0.05), and the L3 group exhibited significantly higher prolactin levels than all short-term antioxidant groups.

### 3.5. Effects of Antioxidants on the Levels of Inflammatory Cytokines in the Sow Placenta

To evaluate whether long-term and short-term antioxidant supplementation could alleviate the inflammatory response of the placenta, we calculated the levels of major inflammatory factors in the placenta in each treatment group. As illustrated in Table 7, the level of the inflammatory cytokine IL-1β in the placentas of all antioxidant-treated groups was significantly decreased compared with the CON group, with the long-term antioxidant groups exhibiting notably lower levels than the short-term antioxidant groups (*p* < 0.05). Similarly, the IL-6 levels in the placentas of all antioxidant-treated groups were significantly reduced compared with the CON group (*p* < 0.05). In contrast, the IL-10 levels in the placentas of the L3 group were significantly increased compared with the CON group, with those in the L3 group being higher than those of all short-term antioxidant groups (*p* < 0.05).

### 3.6. Effects of Antioxidants on Nutrient Transport and Expression Levels of Antioxidant Enzyme-Related Genes in Sow Placenta

**Figure 1:**
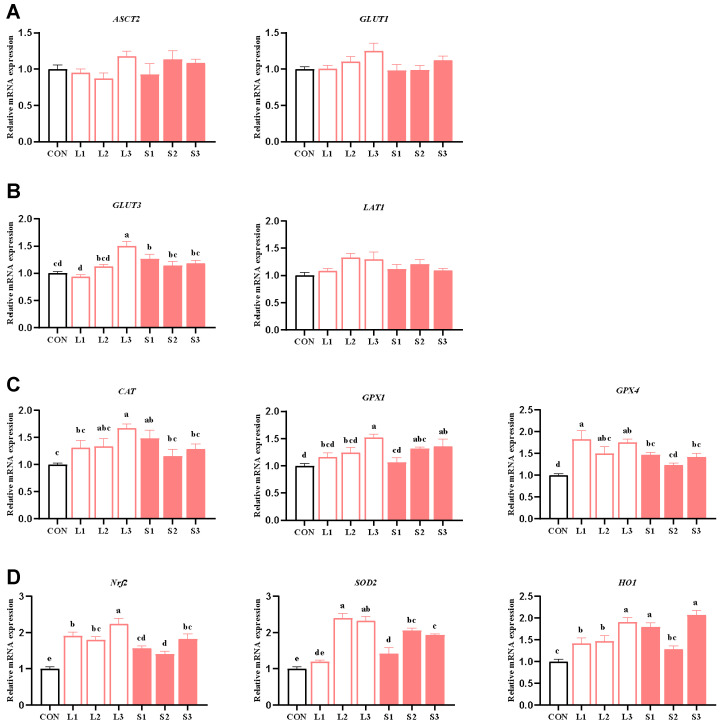
Effects of compound antioxidants on the expression levels of nutrient transport carrier (**A**,**B**) and antioxidant enzyme (**C**,**D**) mRNAs in the placentas of sows. ^a–e^ Different superscript letters indicate significant differences among different treatment groups (*p* < 0.05). CON treatment group: basal diet; L1 treatment group: 25 mg/kg β-carotene added to the basal diet; L2 treatment group: 4 mg/kg SOD added to the basal diet; L3 treatment group: 4 mg/kg SOD added to the basal diet—250 mg/kg combination product was added to the grain; S1 treatment group: 100 mg/kg β-carotene added to the basal diet; S2 treatment group: 14 mg/kg SOD added to the basal diet; S3 treatment group: 1200 mg/kg combination product added to the basal diet; L1–L3: the antioxidant supplementation period began with weaning in the previous breeding cycle and ended with re-breeding after weaning in the present breeding cycle; S1–S3: the antioxidant supplementation period was 7 days before and after weaning in the previous breeding cycle and 7 days before and after farrowing in the present breeding cycle. Data are expressed as means ± SEM, n = 6.

Table 5 and Table 7 show that long-term and short-term antioxidant supplementation can increase the antioxidant enzyme activity of the placenta and alleviate its inflammatory response. We speculated that the placental function may be enhanced. Thus, next, we explored the mechanism behind the placenta’s nutrient transport function and the improvement in the antioxidant capacity. As depicted in Figure 1, compared with the CON group, significant increases (*p* < 0.05) were observed in the mRNA expression levels of *GLUT3* in the L3 and S1 groups; *GPX1* in the L3, S2, and S3 groups; *CAT* in the L3 and S1 groups; *GPX4* in the L1, L2, L3, S1, and S3 groups; *SOD2* in the L2, L3, S1, S2, and S3 groups; *Nrf2* in the L1, L2, L3, S1, S2, and S3 groups; and *HO1* in the L1, L2, L3, S1, and S3 groups. When comparing the long-term and short-term trials, the L3 group showed significantly higher *GLUT3* and *Nrf2* mRNA expression levels than the S1, S2, and S3 groups and higher *CAT* mRNA expression than the S2 and S3 groups. Additionally, the L3 group demonstrated higher *GPX1* and *SOD2* mRNA expression than the S1 group (*p* < 0.05).

### 3.7. Effects of Antioxidants on the Microbial Community Structure in Sow Feces

**Figure 2:**
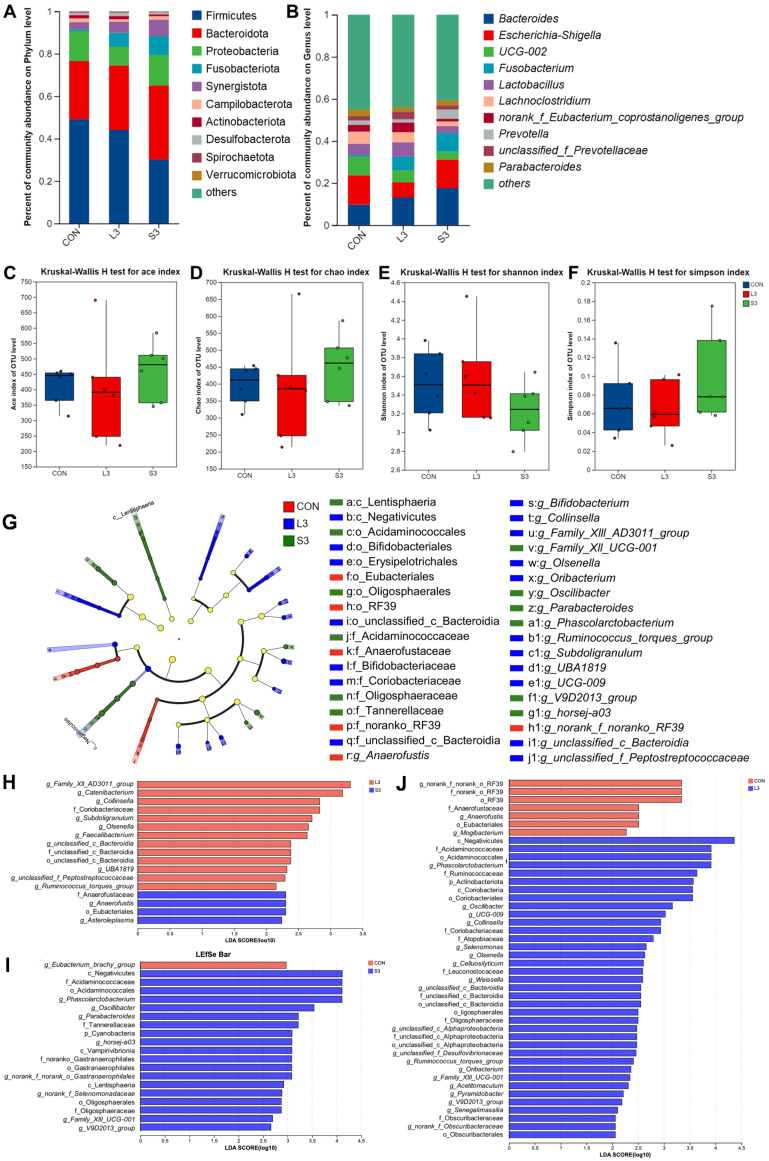
Fecal bacterial community in sows fed diets with complex antioxidant supplementation. Microbial community bar plot of phyla with an abundance of 0.010% or greater (**A**) and microbial community bar plot of genera with a proportion of 0.010% or higher (**B**). The alpha diversity of the fecal bacterial community. ACE index (**C**), CHAO index (**D**), Shannon index (**E**), and Simpson index (**F**) of the sow fecal bacterial community. LEfSe results of the microbiota in fecal samples. Histogram of the linear discriminant analysis scores computed for the differentially abundant features in the fecal bacteria between CON, L3, and S3 treatments (**G**); L3 and S3 treatments (**H**); CON and S3 treatments (**I**); and CON and L3 treatments (**J**). The linear discriminant analysis bars indicate the microbial groups within treatments with linear discriminant analysis scores higher than 2.0. The differentially abundant clades in each treatment are represented by colors in the cladograms, and the linear discriminant analysis scores of these clades indicate the degrees of statistical and biological differences.

Long-term and short-term antioxidant supplementation may have different effects on the composition of the intestinal microbiota, which can affect placental function and fetal development through microbial metabolites. Based on the previous research results, we screened three representative groups, CON, L3, and S3, for amplicon sequencing to explore whether their microbiota structures changed. As depicted in Figure 2, at the phylum level, the gut microbiota of sows was dominated by Firmicutes and Bacteroidota, accounting for over 80% of the total. At the genus level, the microbiota was predominantly composed of Bacteroides, Escherichia-Shigella, UCG-002, Fusobacterium, Lactobacillus, and Lachnoclostridium, collectively accounting for more than 40% of the total gut microbiota. There were no significant differences in microbial diversity and abundance among the treatment groups.

Utilizing principal coordinate analysis (PCoA) based on the unweighted Unifrac and Bray–Curtis metrics, a further clustering analysis was conducted (not displayed). A shorter distribution distance on the coordinate axes indicated a more similar species composition structure, whereas a larger distance represented a greater degree of difference. The results showed that the addition of the compound antioxidants had a significant effect on the intestinal microbial community structure of the sows during farrowing (Adonis; R^2^ = 0.1263, *p* = 0.047).

Figure 2 also presents the LEfSe analysis of the fecal microbiota in sows. Linear discriminant analysis (LDA) was employed to calculate the differences in the abundance of bacterial species at the genus level across various treatment groups, resulting in LDA scores. A higher LDA score indicates a greater impact of the species abundance on the microbial community. The significant differences are visualized as nodes in the cladogram, representing the most discriminant species between the groups. The results show that the CON group was characterized by the presence of g_Anaerofustis as the discriminant species; the L3 treatment group was distinguished by the presence of g_Ruminococcus_torques_group, g_Collinsella, g_Olsenella, g_Subdoligranulum, and g_Oribacterium as the discriminant species; and the S3 treatment group was marked by the presence of g_Phascolarctobacterium, g_Oscillibacter, and g_Parabacteroides as the discriminant species.

### 3.8. Effects of Antioxidants on Growth Performance of Offspring

To investigate whether long-term and short-term antioxidant supplementation could be transmitted from mother to offspring and affect offspring growth and development, we recorded the growth performance of piglets. As indicated in Table 8, the litter weight at weaning and litter weight gain in the L3 group were significantly higher compared with the CON group, with the litter weight gain in the L3 group also notably exceeding those of all short-term antioxidant groups (*p* < 0.05). However, no significant differences were observed in the initial litter weight and diarrhea rate among the groups.

### 3.9. Effects of Antioxidants on Oxidative Stress in Offspring

Table 5 showed that long-term supplementation with compound antioxidants could increase the activity of antioxidant enzymes in the placenta, so we speculated as to whether the antioxidants could affect the antioxidant capacity of the offspring by improving placental function. As shown in Table 9, in the serum of 14 d suckling piglets, the SOD content in the L1, L3, S1, and S3 groups was significantly higher compared with the CON group, with that in the L3 group being higher than in all short-term antioxidant groups (*p* < 0.05). Additionally, the GSH-Px content in the long-term antioxidant groups and the S2 group was significantly elevated compared with the CON group (*p* < 0.05). The NOS content in the L3 group and all short-term antioxidant groups was significantly higher than that in the CON group, and that in the L3 group was higher than that in all short-term antioxidant groups (*p* < 0.05). Furthermore, the MDA content in the L3 group was significantly lower than that in the CON group and all short-term antioxidant groups (*p* < 0.05). In the weaning serum samples of piglets, the T-AOC content in the L3 group was significantly higher than that in the CON group and all short-term antioxidant groups (*p* < 0.05). Compared with the CON group, the GSH-Px content in the L1, L2, and short-term antioxidant groups was significantly increased, and the L2 group had significantly higher GSH-Px content than all short-term antioxidant groups (*p* < 0.05).

### 3.10. Effects of Antioxidants on Serum Growth Hormone Levels in Offspring

Table 8 showed that long-term supplementation with complex antioxidants could improve the litter weight gain in piglets, so we tested the growth hormone levels in piglet serum. As is evident from Table 10, in the serum of weaning piglets, the GH content in the L2, L3, S1, and S3 groups was significantly elevated compared with the CON group (*p* < 0.05), with the L3 group exhibiting significantly higher GH content than all short-term antioxidant groups. However, no significant differences were observed in the serum GH and IGF-1 levels of 14 d suckling piglets or in the IGF-1 content in the weaning serum samples among the various groups.

### 3.11. Effects of Antioxidants on Oxidative Stress and LPS Levels in the Maternal–Fetal Axis in Sows

To determine whether the addition of a composite antioxidant could alleviate maternal oxidative stress and whether LPS was involved in this process, we measured the cortisol levels in the sow serum and placenta, as well as the LPS levels in the maternal–fetal axis. As shown in Table 11, compared with the CON group, the L3 group exhibited significantly decreased cortisol and LPS concentrations on the 40th day of gestation in sows. Furthermore, at farrowing, the cortisol levels in the L1, L2, L3, and S3 groups were significantly reduced (*p* < 0.05). Notably, the placental LPS concentrations in all long-term antioxidant-supplemented groups and the S3 group were significantly decreased compared with the CON group (*p* < 0.05).

### 3.12. Effects of Antioxidants on the Levels of Placental Antioxidant Enzymes and Barrier Function-Related Proteins

**Figure 3:**
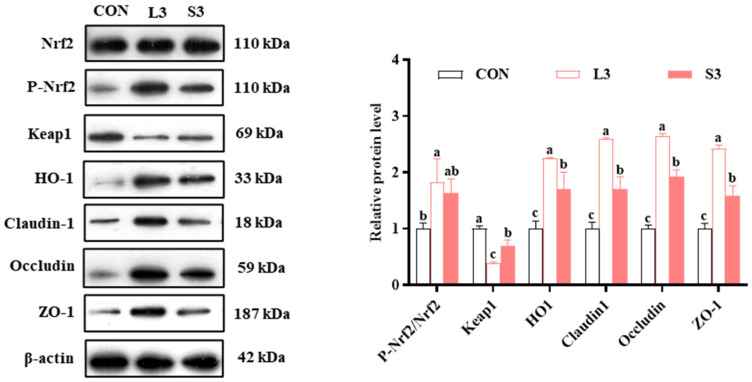
Effects of compound antioxidants on the protein production levels of antioxidant enzymes and barrier function in the placentas of sows. ^a–c^ Different superscript letters indicate significant differences among different treatment groups (*p* < 0.05). CON treatment group: basal diet; L3 treatment group: 4 mg/kg SOD added to the basal diet—250 mg/kg combination product was added to the grain; S3 treatment group: 1200 mg/kg combination product added to the basal diet; L3: the antioxidant supplementation period began with weaning in the previous breeding cycle and ended with re-breeding after weaning in the present breeding cycle; S3: the antioxidant supplementation period was 7 days before and after weaning in the previous breeding cycle and 7 days before and after farrowing in the present breeding cycle. Data are expressed as means ± SEM, n = 3.

The qPCR results indicated that long-term supplementation with a composite antioxidant enhanced the expression of nutrient transport carriers and antioxidant pathway-related genes in the placentas of sows. Therefore, we subsequently examined the levels of intestinal barrier- and antioxidant pathway-related proteins in the placenta. The results regarding protein production are presented in Figure 3. Regarding the production of antioxidant enzymes, the L3 group had significantly increased P-Nrf2/Nrf2 and HO1 levels and significantly decreased Keap1 levels compared with the CON group. Similarly, the S3 group had significantly upregulated HO1 and downregulated Keap1 (*p* < 0.05). In terms of barrier function, both the L3 and S3 groups showed significantly higher levels of Claudin1, Occludin, and ZO-1 compared with the CON group; notably, those in the L3 group were higher than in the S3 group (*p* < 0.05).

## 4. Discussion

Gestating and lactating sows are susceptible to oxidative stress, which can damage their tissues and cells, ultimately leading to reduced reproductive efficiency [26,27]. While numerous studies have investigated the feasibility of antioxidants, limited research has focused on the specific supplementation strategies and the transmission of antioxidant capacity between sows and their offspring. From the perspective of mitigating oxidative stress, an innovative approach was undertaken by combining β-carotene and SOD to enhance the health status of both sows and piglets. Our findings reveal that the long-term, low-dose supplementation of these two antioxidants during the gestation and lactation of sows can reduce oxidative stress, and, importantly, this beneficial effect is transmitted through the placenta and colostrum, ultimately contributing to enhanced efficiency in piglet–sow integrated production systems.

### 4.1. Multi-Component Antioxidants Enhanced Sow Reproductive Performance by Modulating Oxidative Stress, Inflammatory Responses, and Gut Microbiota Composition

Because oxidative stress is a key factor in reproductive performance, improving the antioxidant capacity of sows is important [28]. Our study revealed that supplementation with β-carotene and SOD, whether administered in long-term low concentrations or short-term high concentrations, effectively improved the antioxidant indices in the serum, colostrum, and placentas of sows. Notably, long-term combined supplementation emerged as the most effective approach. This efficacy can be attributed to the complementary nature of β-carotene, a precursor of vitamin A that non-enzymatically scavenges free radicals [29], and SOD, an antioxidant metalloenzyme that is inherently present in biological systems [19]. Collectively, they synergistically augment the overall antioxidant defense system through two distinct pathways [18,20]. In this study, there was no significant difference in backfat loss between the L1 and L2 groups and the control group, while the backfat loss of the L3 group was significantly reduced, indicating that the combined use of the antioxidants had a better effect. In addition, the reduction in H_2_O_2_ and IL-1β in the L3 group may also explain this phenomenon. The data in Table 5 and Table 7 support this. Additionally, physiological levels of free radicals are essential for various biochemical processes, although their excess can lead to detrimental effects. Therefore, a balance between the oxidative and antioxidant status is vital in achieving steady-state free radical levels [30]. Long-term low-dose supplementation potentially enables the maintenance of a stable and appropriate antioxidant capacity in sows, thereby enhancing the overall antioxidant efficacy and reproductive performance.

Inflammatory responses are often intricately linked with oxidative stress and are significantly modulated by the LPS levels [31,32]. Oxidative stress refers to the damaging and stressful effects that oxygen-derived free radicals and other ROS exert on cells within and outside the cellular environment. These ROS can activate inflammatory pathways, leading to the infiltration of inflammatory cells and the release of inflammatory mediators [27]. LPS, consisting of macromolecules predominantly found in the outer membranes of Gram-negative bacteria, is released post-bacterial cell death, traverses the intestinal barrier, and enters the bloodstream, potentially propagating to distant organs to induce inflammatory responses. In this study, the supplementation of antioxidants resulted in the decreased expression of pro-inflammatory cytokines IL-1β and IL-6, indicating the mitigation of inflammatory responses, which may be correlated with a decrease in circulating LPS levels [33]. In addition, the fact that the addition of two antioxidants at the same time, as shown in Table 7, leads to increased IL-6 secretion may reflect the activation of immunity rather than a pathological response [34]. The inflammatory cascade triggered by LPS involves the activation of immune cells, resulting in the release of oxygen free radicals and other inflammatory mediators, thereby enhancing oxidative stress. This finding is supported by the improvement observed in the blood cortisol levels and other antioxidant indices. While the reduction in IL-1β and IL-6 might superficially suggest dampened immune defenses, our data argue against this interpretation. Antioxidant supplementation concurrently elevated the levels of anti-inflammatory IL-10 and immunoglobulins (IgG/IgM), indicating a balanced immune response rather than suppression. Critically, the placental LPS levels decreased in the treated groups, likely due to gut microbiota shifts (e.g., the suppression of Escherichia-Shigella) and enhanced intestinal barrier function. No clinical infections were observed, aligning with evidence that redox balance supports—rather than compromises—host defense. Notably, IgG did not continue to increase in the S3 group, suggesting a potential ceiling effect in immunoglobulin synthesis, where combined antioxidants may activate overlapping pathways or compete for metabolic resources.

An expanding research corpus has emphasized the capacity of antioxidants to augment sow reproductive performance through the modulation of the gut microbiota. Our data indicate that antioxidant supplementation led to the enrichment of several beneficial bacteria. Specifically, Subdoligranulum, which ferments glucose and other sugars to produce acetate and succinate, exhibited a positive correlation with the daily weight gain and fiber utilization efficiency in pigs, as evidenced by a prior study [35]. Bifidobacterium, widely recognized as a probiotic, modulates diverse functions including nutrient metabolism and immune modulation. Its increased abundance is often associated with modulated gut conditions and enhanced productivity in sows [36,37]. Furthermore, Olsenella, a prevalent beneficial component of the gut microbiota, plays a pivotal role in maintaining intestinal health and facilitating nutrient absorption [38]. Parabacteroides, primarily colonizing the gut, demonstrates the capability to degrade fiber into acetate and propionate, while its secretions, like ursodeoxycholic acid, aid the host in metabolic regulation and anti-inflammatory processes [39]. Collectively, these findings demonstrate that the elevation of the antioxidant capacity, alleviation of inflammatory responses, and optimization of the gut microbiota structure reflect the cumulative benefits of long-term antioxidant combination therapy. This improvement in the sows’ health status may have contributed to the increased litter size at weaning and reduced backfat loss, highlighting the potential of antioxidant interventions in enhancing the integrated production of sows and piglets.

### 4.2. Synergistic Mechanisms Underlying Antioxidant Efficacy

The administration of β-carotene and SOD synergistically improved sow and piglet health through multifaceted mechanisms. In the mammary gland, increased milk protein synthesis likely resulted from the antioxidant protection of mammary epithelial cells (MECs), where β-carotene scavenged lipid peroxides to preserve the protein synthesis machinery, while SOD maintained ATP production for casein synthesis [40]. Elevated prolactin further amplified milk protein gene expression via enhanced receptor signaling. Hormonal modulation involved reduced oxidative stress stabilizing ovarian luteal cells, thereby sustaining progesterone synthesis, while the SOD-mediated suppression of hypothalamic ROS improved dopamine turnover, disinhibiting prolactin and GH secretion. Concurrently, lowered systemic LPS and IL-1β reduced hypothalamic–pituitary–adrenal (HPA) axis activation, decreasing cortisol [41]. Enhanced barrier integrity was driven by ROS suppression stabilizing tight junction proteins (Claudin1, Occludin, ZO-1) via cytoskeletal protection and NF-κB inhibition, alongside Nrf2 activation by β-carotene [42]. Placental nutrient transport was improved via GLUT3 upregulation, where antioxidants stabilized HIF-1α to enhance glucose uptake in trophoblasts and supported fetal capillary development through reduced IL-6 [43]. These interconnected mechanisms—spanning redox balance, hormonal regulation, barrier function, and metabolic adaptation—collectively enhanced sows’ reproductive performance and piglets’ growth.

### 4.3. Multi-Component Antioxidants in Sow Feed Enhanced Piglets’ Growth Performance by Improving Nutrient Delivery and the Overall Health Status

The placenta, serving as a vital interface connecting and separating the mother and fetus during pregnancy, is a key factor for reproductive performance [44]. It mediates bidirectional exchanges between the maternal and fetal circulation, encompassing the provision of nutrients and oxygen to the fetus and the excretion of metabolic waste back to the mother. Additionally, it acts as a protective barrier against pathogens, ensuring an optimal intrauterine environment for fetal prenatal development and maternal health [45,46]. In the present study, the appropriate use of antioxidants enhanced the expression of genes and proteins associated with nutrient transport and barrier function in the placenta, suggesting the enhanced and efficient transfer of nutrients from the sow to the fetus and the effective protection of the fetus from harmful substances. In addition, progesterone levels are critical for the maintenance of placental development, and the results of this study suggest that the synergistic effects of β-carotene and SOD in L3 may stabilize the redox balance, thereby supporting progesterone synthesis, while low-dose intervention alone (L1/L2) is insufficient to offset oxidative stress. The colostrum, the first source of nutrients and immunity for piglets, exhibited increased levels of milk proteins and immunoglobulins in the antioxidant-treated group. This enhancement fortifies piglets’ ability to acquire sufficient nutrition and resist diseases immediately after birth.

The neonatal piglet phase serves as a crucial foundation for weight gain in later life stages [47]. Nursing pigs are susceptible to oxidative stress due to rapid growth and development, characterized by swift cell division and proliferation and their vigorous metabolism [48]. In the current study, the appropriate inclusion of antioxidants in sow diets effectively bolstered the antioxidant capacity of piglets at both 14 days of lactation and weaning, reflecting the transmission of antioxidant capacity from sows to piglets, consistent with previous research findings [49]. While the gut microbiota of piglets was not directly examined, extensive research has established that the fecal microbiota of sows influences gut microbiota homeostasis in piglets [50]. Consequently, the abundant beneficial bacteria present in the antioxidant-treated sow groups may have contributed to enhanced nutrient metabolism and inflammation suppression in piglets. In conclusion, the appropriate use of antioxidants in sow diets improves nutrient provisioning to nursing piglets and elevates their antioxidant defenses, ultimately resulting in significant gains in piglet weight during lactation and increased litter weight at weaning.

### 4.4. The Placenta and Colostrum May Serve as Pivotal Pathways for the Transmission of Antioxidant Capacity

Oxidative stress during gestation and lactation in sows can have severe detrimental effects on the growth and development of offspring, particularly impacting their antioxidant capacity [51]. Firstly, during early embryogenesis, oxidative stress can disrupt normal cell division and development by damaging the DNA, proteins, and lipids of embryonic cells. Then, it can interfere with the nutrient supply to the embryo by compromising placental function [52]. Furthermore, placental inflammation and impaired barrier function induced by oxidative stress allow harmful substances such as endotoxins to enter the fetal circulation [9]. The first few days post-birth are crucial for piglets to establish a robust physical foundation and antioxidant capacity, which is predicated on the abundant nutrients, immune factors, and antioxidant capacity regulators present in colostrum [53]. In summary, previous findings suggest that the placenta and colostrum may serve as pivotal conduits for the transfer of antioxidant capacity, a hypothesis that is also supported by the data from the present study.

In the present study, the reasonable use of antioxidants effectively mitigated oxidative stress and reduced the inflammatory responses in the placenta, thereby providing a low-stress environment conducive to fetal growth. Additionally, the decrease in LPS levels and enhancement in barrier function in the placenta minimized the influx of oxidative stressors into the fetus. Adequate nutrient availability is fundamental to the realization of physiological functions, and the rational application of antioxidants facilitated the optimal transfer of nutrients via the placenta during gestation and through the colostrum postnatally, thereby enhancing the likelihood of piglets obtaining sufficient nutrients. Furthermore, the elevated levels of antioxidant capacity regulators and immunoglobulins in the colostrum also contributed to the enhanced antioxidant status of the piglets.

## 5. Conclusions

Multi-component antioxidant blend supplementation at a low dose throughout the gestation and lactation stages effectively enhanced the antioxidant capacity of sows during gestation and lactation, ameliorated placental inflammatory responses, optimized nutrient transfer and barrier functions, and reduced the likelihood of endotoxin transmission to the fetus. These changes may have been associated with the enhanced antioxidant and growth performance observed in the piglets. These findings provide novel insights into the transfer of antioxidant capacity between the maternal and fetal compartments and offer a technical solution for the efficient integrated management of sows and piglets.

## Figures and Tables

**Table 1:** Ingredients and nutrient compositions of basal diets (as-fed basis).

Item	Gestation Diet	Lactation Diet
Ingredients, %		
Corn	46.50	59.00
Wheat feed flour	5.00	5.00
Wheat bran	22.00	8.00
Rice bran	10.00	-
Defatted rice bran	8.00	-
Soybean meal	5.00	11.00
Expanded soybean	-	10.00
Fish meal	-	2.00
Soybean oil	0.50	1.00
NaCl	0.50	-
Limestone	1.50	-
Premix ^1^	1.00	4.00
Nutrient levels		
Digestible energy, Kcal/kg	3030	3335
Metabolizable energy, Kcal/kg	2897	3150
Net energy, Kcal/kg	2254	2455
Crude protein, %	12.18	16.00
Crude fiber, %	4.58	3.04
Total calcium, %	0.67	0.88
Total phosphorus, %	0.68	0.59
Total lysine, %	0.65	1.03

^1^ Provided per kilogram of diet: Vitamin A, 12,000 IU; Vitamin D3, 2000 IU; Vitamin E, 25 IU; Vitamin K3, ≥2 mg; Vitamin B1, ≥2 mg; Vitamin B2, ≥6 mg; Vitamin B6, ≥4 mg; Vitamin B12, ≥24 ng; Niacin, 30 mg; Pantothenic acid, ≥20 mg; Folic acid, ≥3.6 mg; Biotin, ≥0.4 mg; Fe, 96 mg; Cu, 8.0 mg; Zn, 120 mg; Mn, 40 mg; I, 0.56 mg; Se, 0.4 mg.

**Table 2:** The primer sequences of the target genes used for RT-qPCR.

Gene Name	Forward Primer Sequence (5′-3′)	Reverse Primer Sequence (5′-3′)	Accession Number
*ASCT2*	CATCAGCCGCTTCATTCTGC	GACTGCCTCGAGGATGATGG	XM_003355984
*GLUT1*	CGTCGCTGGCTTCTCCAACT	CCAGGAGCACCGTGAAGATGAT	XM_021096908
*GLUT3*	TCTTGGTCTTCGTGGCCTTC	GACAACGAGGAAGCAGGTGA	XM_021092392
*LAT1*	GCCCATTGTCACCATCATC	GAGCCCACAAAGAAAAGC	NM_001110421
*CAT*	ACGCCTGTGTGAGAACATTG	GTCCAGAAGAGCCTGAATGC	NM_214301
*GPX1*	AAATGCTCACCCGCTCTTC	GTCATTGCGACACACTGGAG	NM_214201
*GPX4*	CACCCTCTGTGGAAGTGGAT	TCACCACACAGCCGTTCTTA	NM_214407
*SOD2*	GACCTGCCGTACGACTATGG	TCAGGTTGTTCACGTAGGCC	NM_214127
*Nrf2*	GGGCCCATTGATCTCTCTGA	GAAGCCAAGCAGTGTGTCTC	NM_001114671
*HO1*	ACAGAAGAGGCTAAGACCG	CAGGCATCTCCTTCCATT	NM_001004027

*ASCT2* = Alanine-Serine-Cysteine Transporter 2; *GLUT1* = Glucose Transporter 1; *GLUT3* = Glucose Transporter 3; *LAT1* = L-Type Amino Acid Transporter 1; *CAT* = Catalase; *GPX1* = Glutathione Peroxidase 1; *GPX4* = Glutathione Peroxidase 4; *SOD2* = Superoxide Dismutase 2; *Nrf2* = Nuclear Factor Erythroid 2-Related Factor 2; *HO1* = Heme Oxygenase 1.

**Table 3:** Effects of compound antioxidant supplementation on production performance of sows.

Item	CON	L1	L2	L3	S1	S2	S3	SEM	*p* Value
Total number of piglets born, n	13.79	14.55	14.00	14.89	14.10	14.22	14.84	0.22	0.79
Number of piglets born alive, n	13.32	13.95	13.17	14.32	13.50	13.72	13.89	0.24	0.90
Number of healthy piglets, n	12.79	13.20	12.72	13.32	12.75	13.06	12.89	0.26	0.99
Number of stillbirths, n	0.47	0.60	0.83	0.58	0.60	0.50	0.95	0.08	0.71
Number of mummies, n	0.26	0.25	0.33	0.05	0.20	0.17	0.32	0.04	0.58
Number of piglets weaned, n	12.53 ^bc^	13.15 ^ab^	12.22 ^c^	13.53 ^a^	12.45 ^bc^	12.67 ^bc^	13.05 ^ab^	0.10	<0.05
Farrowing interval, min	310.53	314.50	319.44	273.16	278.00	312.22	308.95	5.03	0.33
Backfat thickness loss, mm	3.42 ^a^	3.50 ^a^	3.22 ^ab^	2.79 ^b^	2.95 ^b^	3.06 ^ab^	3.47 ^a^	0.06	<0.05
Wean-to-estrus interval, d	4.95	4.93	5.06	4.97	5.03	4.92	4.87	0.05	0.97

Note: ^a–c^ Different superscript letters indicate significant differences among different treatment groups (*p* < 0.05). CON treatment group: basal diet; L1 treatment group: 25 mg/kg β-carotene added to the basal diet; L2 treatment group: 4 mg/kg SOD added to the basal diet; L3 treatment group: a mixture of 25 mg/kg β-carotene and 4 mg/kg SOD added to the basal diet; S1 treatment group: 100 mg/kg β-carotene added to the basal diet; S2 treatment group: 14 mg/kg SOD added to the basal diet; S3 treatment group: a mixture of 100 mg/kg β-carotene and 14 mg/kg SOD added to the basal diet; L1–L3: the antioxidant supplementation period began with weaning in the previous breeding cycle and ended with re-breeding after weaning in the present breeding cycle; S1–S3: the antioxidant supplementation period was 7 days before and after weaning in the previous breeding cycle and 7 days before and after farrowing in the present breeding cycle. SEM = pooled standard error of the mean. n = 18–20.

**Table 4:** Effects of compound antioxidant supplementation on colostrum composition and immunoglobulin levels of sows.

Item	CON	L1	L2	L3	S1	S2	S3	SEM	*p* Value
Composition									
Milk fat, %	6.31	6.66	6.23	6.15	6.27	6.55	6.32	0.05	0.06
Milk protein, mg/mL	4.90 ^c^	5.10 ^cb^	5.40 ^b^	5.79 ^a^	5.70 ^a^	4.82 ^c^	4.90 ^c^	0.06	<0.05
Lactose, mg/mL	20.67	21.61	20.74	21.47	21.09	20.53	21.20	0.22	0.83
Total solids, %	13.18	12.51	12.42	13.24	12.23	12.92	12.60	0.15	0.49
Immunoglobulin, μg/mL									
IgA	37.30	37.07	38.06	37.1	36.93	38.31	35.84	0.28	0.31
IgG	348.50 ^c^	355.50 ^c^	396.80 ^a^	399.10 ^a^	400.90 ^a^	379.10 ^b^	374.50 ^b^	3.22	<0.05
IgM	39.25 ^c^	39.88 ^bc^	44.17 ^a^	43.17 ^ab^	41.30 ^ac^	40.06 ^c^	41.99 ^ac^	0.38	<0.05

Note: ^a–c^ Different superscript letters indicate significant differences among different treatment groups (*p* < 0.05). CON treatment group: basal diet; L1 treatment group: 25 mg/kg β-carotene added to the basal diet; L2 treatment group: 4 mg/kg SOD added to the basal diet; L3 treatment group: a mixture of 25 mg/kg β-carotene and 4 mg/kg SOD added to the basal diet; S1 treatment group: 100 mg/kg β-carotene added to the basal diet; S2 treatment group: 14 mg/kg SOD added to the basal diet; S3 treatment group: a mixture of 100 mg/kg β-carotene and 14 mg/kg SOD added to the basal diet; L1–L3: the antioxidant supplementation period began with weaning in the previous breeding cycle and ended with re-breeding after weaning in the present breeding cycle; S1–S3: the antioxidant supplementation period was 7 days before and after weaning in the previous breeding cycle and 7 days before and after farrowing in the present breeding cycle. SEM = pooled standard error of the mean. n = 6.

**Table 5:** Effects of antioxidants on antioxidant capacity indicators in blood, colostrum, and placentas of sows.

Item	CON	L1	L2	L3	S1	S2	S3	SEM	*p* Value
Serum of sows at 40 days of gestation									
T-AOC, μmol/mL	3.14	3.20	3.15	3.20	3.14	3.15	3.19	0.03	0.99
SOD, U/L	1493.83 ^c^	1457.17 ^d^	1505.27 ^c^	1622.91 ^a^	1596.80 ^b^	1420.25 ^e^	1487.14 ^c^	11.01	<0.05
GSH-Px, IU/L	151.40	146.94	145.75	149.11	151.56	148.82	152.02	0.71	0.11
NOS, U/mL	1.72 ^e^	1.97 ^b^	2.09 ^a^	1.71 ^e^	1.77 ^d^	1.86 ^c^	1.95 ^b^	0.02	<0.05
H_2_O_2_, μmol/L	25.29 ^bc^	27.64 ^a^	21.56 ^e^	24.42 ^d^	25.81 ^b^	25.00 ^cd^	21.13 ^e^	0.35	<0.05
MDA, nmol/L	8.86	8.50	8.73	8.46	8.42	8.81	8.52	0.15	0.20
Serum of sows at farrowing									
T-AOC, μmol/mL	5.14 ^b^	5.17 ^b^	5.27 ^ab^	5.42 ^a^	5.22 ^ab^	5.15 ^b^	5.25 ^ab^	0.02	<0.05
SOD, U/L	2187.78	2171.42	2187.82	2110.25	2197.35	2169.01	2114.28	11.30	0.19
GSH-Px, IU/L	253.36	249.10	250.12	247.68	245.10	244.69	255.16	1.35	0.30
NOS, U/mL	2.77 ^e^	2.97 ^b^	2.81 ^e^	2.88 ^d^	2.93 ^c^	2.98 ^b^	3.07 ^a^	0.02	<0.05
H_2_O_2_, μmol/L	42.43 ^b^	43.69 ^a^	39.80 ^de^	39.16 ^e^	40.75 ^c^	39.88 ^d^	39.33 ^de^	0.26	<0.05
MDA, nmol/L	13.02	12.80	13.04	12.72	12.48	12.51	12.62	0.16	0.18
Serum of sows at weaning									
T-AOC, μmol/mL	9.02	9.08	8.94	9.06	8.99	9.09	9.00	0.04	0.96
SOD, U/L	1747.28 ^b^	1689.99 ^c^	1674.59 ^c^	1821.86 ^a^	1742.36 ^b^	1658.67 ^c^	1758.02 ^b^	9.35	<0.05
GSH-Px, IU/L	203.92	204.43	202.63	204.17	199.91	198.73	204.46	0.57	0.10
NOS, U/mL	2.10 ^e^	2.13 ^d^	2.14 ^d^	2.41 ^a^	2.19 ^c^	2.35 ^b^	2.18 ^c^	0.02	<0.05
H_2_O_2_, μmol/L	30.88	30.04	30.80	29.42	29.38	30.56	30.03	0.40	0.93
MDA, nmol/L	10.55	9.99	10.74	10.59	10.62	10.30	10.60	0.08	0.16
Colostrum									
T-AOC, μmol/mL	10.64 ^bc^	10.62 ^c^	10.50 ^c^	10.74 ^bc^	10.68 ^bc^	10.91 ^b^	11.27 ^a^	0.04	<0.05
SOD, U/L	2020.15	2011.45	2032.43	2018.24	1995.16	1983.60	2032.99	7.58	0.56
GSH-Px, IU/L	169.64 ^f^	185.18 ^d^	188.74 ^b^	186.11 ^cd^	191.12 ^a^	188.03 ^bc^	177.10 ^e^	1.14	<0.05
NOS, U/mL	2.59	2.59	2.60	2.56	2.55	2.58	2.61	0.01	0.74
H_2_O_2_, μmol/L	32.56	33.37	33.13	31.41	30.61	29.60	30.49	0.52	0.36
MDA, nmol/L	12.27	12.02	12.09	11.83	12.21	12.53	11.99	0.11	0.70
Placenta									
T-AOC, μmol/mL	78.16 ^b^	83.78 ^a^	78.83 ^b^	84.75 ^a^	78.09 ^b^	84.67 ^a^	75.65 ^b^	0.63	<0.05
SOD, U/L	1954.61 ^b^	1974.25 ^ab^	1805.37 ^d^	2005.25 ^a^	1806.00 ^d^	1905.39 ^c^	1743.01 ^e^	15.10	<0.05
GSH-Px, IU/L	224.40	219.63	217.56	226.52	217.57	216.97	213.81	1.32	0.12
NOS, U/mL	2.00 ^de^	1.91 ^f^	2.21 ^a^	2.16 ^c^	2.00 ^d^	2.07 ^c^	1.95 ^ef^	0.02	<0.05
H_2_O_2_, μmol/L	373.28 ^a^	352.52 ^b^	340.36 ^cd^	338.76 ^d^	354.58 ^b^	345.86 ^c^	338.28 ^d^	1.94	<0.05
MDA, nmol/L	10.73 ^a^	10.76 ^a^	10.67 ^a^	9.87 ^c^	10.08 ^bc^	10.59 ^ab^	10.55 ^ab^	0.08	<0.05

Note: ^a–f^ Different superscript letters indicate significant differences among different treatment groups (*p* < 0.05). CON treatment group: basal diet; L1 treatment group: 25 mg/kg β-carotene added to the basal diet; L2 treatment group: 4 mg/kg SOD added to the basal diet; L3 treatment group: a mixture of 25 mg/kg β-carotene and 4 mg/kg SOD added to the basal diet; S1 treatment group: 100 mg/kg β-carotene added to the basal diet; S2 treatment group: 14 mg/kg SOD added to the basal diet; S3 treatment group: a mixture of 100 mg/kg β-carotene and 14 mg/kg SOD added to the basal diet; L1–L3: the antioxidant supplementation period began with weaning in the previous breeding cycle and ended with re-breeding after weaning in the present breeding cycle; S1–S3: the antioxidant supplementation period was 7 days before and after weaning in the previous breeding cycle and 7 days before and after farrowing in the present breeding cycle. SEM = pooled standard error of the mean. n = 6.

**Table 6:** Effects of compound antioxidant supplementation on serum hormone levels of sows.

Item	CON	L1	L2	L3	S1	S2	S3	SEM	*p* Value
Progesterone concentration at day 40 of gestation, pmol/L	2218.02 ^d^	2146.68 ^d^	2131.58 ^d^	3174.40 ^a^	2473.65 ^c^	2174.17 ^d^	2534.89 ^b^	64.77	<0.05
Prolactin concentration at the time of farrowing, ng/L	223.45 ^e^	302.85 ^b^	305.34 ^b^	312.90 ^a^	199.64 ^f^	236.39 ^d^	280.73 ^c^	6.59	<0.05

Note: ^a–f^ Different superscript letters indicate significant differences among different treatment groups (*p* < 0.05). CON treatment group: basal diet; L1 treatment group: 25 mg/kg β-carotene added to the basal diet; L2 treatment group: 4 mg/kg SOD added to the basal diet; L3 treatment group: a mixture of 25 mg/kg β-carotene and 4 mg/kg SOD added to the basal diet; S1 treatment group: 100 mg/kg β-carotene added to the basal diet; S2 treatment group: 14 mg/kg SOD added to the basal diet; S3 treatment group: a mixture of 100 mg/kg β-carotene and 14 mg/kg SOD added to the basal diet; L1–L3: the antioxidant supplementation period began with weaning in the previous breeding cycle and ended with re-breeding after weaning in the present breeding cycle; S1–S3: the antioxidant supplementation period was 7 days before and after weaning in the previous breeding cycle and 7 days before and after farrowing in the present breeding cycle. SEM = pooled standard error of the mean. n = 6.

**Table 7:** Effects of compound antioxidant supplementation on inflammatory cytokine levels in placentas of sows.

Item	CON	L1	L2	L3	S1	S2	S3	SEM	*p* Value
Interleukin-1β, ng/L	44.72 ^a^	34.19 ^d^	31.18 ^e^	29.86 ^f^	41.55 ^b^	41.96 ^b^	39.28 ^c^	0.85	<0.05
Interleukin-6, ng/L	982.19 ^a^	663.00 ^e^	816.11 ^c^	882.19 ^b^	741.15 ^d^	618.81 ^f^	816.64 ^c^	18.20	<0.05
Interleukin-10, ng/L	159.67 ^c^	130.49 ^e^	152.85 ^d^	183.78 ^a^	172.30 ^b^	123.85 ^f^	174.82 ^b^	9.30	<0.05
Tumor necrosis factor-α, pg/mL	255.66	256.89	246.92	259.23	252.48	261.89	260.92	1.73	0.25

Note: ^a–f^ Different superscript letters indicate significant differences among different treatment groups (*p* < 0.05). CON treatment group: basal diet; L1 treatment group: 25 mg/kg β-carotene added to the basal diet; L2 treatment group: 4 mg/kg SOD added to the basal diet; L3 treatment group: a mixture of 25 mg/kg β-carotene and 4 mg/kg SOD added to the basal diet; S1 treatment group: 100 mg/kg β-carotene added to the basal diet; S2 treatment group: 14 mg/kg SOD added to the basal diet; S3 treatment group: a mixture of 100 mg/kg β-carotene and 14 mg/kg SOD added to the basal diet; L1–L3: the antioxidant supplementation period began with weaning in the previous breeding cycle and ended with re-breeding after weaning in the present breeding cycle; S1–S3: the antioxidant supplementation period was 7 days before and after weaning in the previous breeding cycle and 7 days before and after farrowing in the present breeding cycle. SEM = pooled standard error of the mean. n = 6.

**Table 8:** Effects of compound antioxidant supplementation on the growth performance of suckling piglets.

Item	CON	L1	L2	L3	S1	S2	S3	SEM	*p* Value
Litter weight at farrowing, kg	19.86	21.04	19.91	19.83	19.56	19.02	20.77	0.36	0.78
Litter weight at weaning, kg	91.00 ^b^	97.83 ^ab^	91.14 ^b^	100.05 ^a^	91.80 ^ab^	92.31 ^ab^	96.08 ^ab^	0.81	<0.05
Litter weight gain from farrowing to weaning, kg	71.14 ^b^	76.79 ^ab^	71.23 ^b^	80.22 ^a^	72.24 ^b^	73.28 ^b^	75.31 ^ab^	0.65	<0.05
Diarrhea incidence, %	8.20	7.76	8.75	7.11	8.49	7.90	7.53	0.23	0.52

Note: ^a,b^ Different superscript letters indicate significant differences among different treatment groups (*p* < 0.05). CON treatment group: basal diet; L1 treatment group: 25 mg/kg β-carotene added to the basal diet; L2 treatment group: 4 mg/kg SOD added to the basal diet; L3 treatment group: a mixture of 25 mg/kg β-carotene and 4 mg/kg SOD added to the basal diet; S1 treatment group: 100 mg/kg β-carotene added to the basal diet; S2 treatment group: 14 mg/kg SOD added to the basal diet; S3 treatment group: a mixture of 100 mg/kg β-carotene and 14 mg/kg SOD added to the basal diet; L1–L3: the antioxidant supplementation period began with weaning in the previous breeding cycle and ended with re-breeding after weaning in the present breeding cycle; S1–S3: the antioxidant supplementation period was 7 days before and after weaning in the previous breeding cycle and 7 days before and after farrowing in the present breeding cycle. SEM = pooled standard error of the mean. n = 20.

**Table 9:** Effects of compound antioxidant supplementation on antioxidant capacity of piglets.

Item	CON	L1	L2	L3	S1	S2	S3	SEM	*p* Value
Piglets at d 14									
T-AOC, μmol/mL	9.45	9.54	9.43	9.42	9.44	9.31	9.38	0.04	0.87
SOD, U/L	1871.06 ^d^	1929.01 ^b^	1800.54 ^e^	1990.32 ^a^	1897.67 ^c^	1764.19 ^f^	1949.16 ^b^	11.96	<0.05
GSH-Px, IU/L	194.38 ^e^	205.77 ^b^	197.71 ^d^	202.76 ^c^	192.46 ^e^	207.95 ^a^	190.08 ^f^	3.03	<0.05
NOS, U/mL	1.97 ^e^	1.92 ^f^	1.98 ^e^	2.23 ^a^	2.04 ^d^	2.09 ^c^	2.13 ^b^	0.02	<0.05
H_2_O_2_, μmol/L	28.23	29.47	27.65	27.11	30.05	29.30	30.01	0.37	0.19
MDA, nmol/L	9.06 ^d^	9.96 ^a^	9.95 ^a^	8.80 ^e^	9.53 ^b^	9.29 ^c^	9.58 ^b^	0.06	<0.05
Piglets at weaning									
T-AOC, μmol/mL	9.55 ^b^	9.68 ^ab^	9.15 ^c^	9.93 ^a^	8.59 ^d^	9.15 ^c^	9.43 ^b^	0.37	<0.05
SOD, U/L	1765.01	1740.35	1773.07	1738.35	1708.77	1781.59	1711.40	7.55	0.12
GSH-Px, IU/L	188.30 ^e^	193.28 ^d^	208.22 ^a^	189.43 ^e^	198.54 ^c^	201.88 ^b^	193.69 ^d^	1.06	<0.05
NOS, U/mL	2.26	2.22	2.24	2.26	2.21	2.28	2.23	0.01	0.77
H_2_O_2_, μmol/L	30.21	30.29	29.79	30.46	30.33	30.53	30.74	0.35	0.99
MDA, nmol/L	9.56 ^d^	10.23 ^b^	9.47 ^e^	9.75 ^cd^	9.85 ^c^	10.46 ^a^	10.54 ^a^	0.16	<0.05

Note: ^a–f^ Different superscript letters indicate significant differences among different treatment groups (*p* < 0.05). CON treatment group: basal diet; L1 treatment group: 25 mg/kg β-carotene added to the basal diet; L2 treatment group: 4 mg/kg SOD added to the basal diet; L3 treatment group: a mixture of 25 mg/kg β-carotene and 4 mg/kg SOD added to the basal diet; S1 treatment group: 100 mg/kg β-carotene added to the basal diet; S2 treatment group: 14 mg/kg SOD added to the basal diet; S3 treatment group: a mixture of 100 mg/kg β-carotene and 14 mg/kg SOD added to the basal diet; L1–L3: the antioxidant supplementation period began with weaning in the previous breeding cycle and ended with re-breeding after weaning in the present breeding cycle; S1–S3: the antioxidant supplementation period was 7 days before and after weaning in the previous breeding cycle and 7 days before and after farrowing in the present breeding cycle. SEM = pooled standard error of the mean. n = 6.

**Table 10:** Effects of compound antioxidant supplementation on serum hormone levels in suckling piglets.

Item	CON	L1	L2	L3	S1	S2	S3	SEM	*p* Value
Piglets at d 14, μg/L									
GH	35.34	36.35	34.38	35.27	34.33	35.42	34.60	0.47	0.92
IGF-1	17.24	17.70	18.18	17.15	17.25	18.22	18.24	0.16	0.18
Piglets at weaning, μg/L									
GH	34.10 ^b^	33.71 ^b^	36.94 ^a^	37.35 ^a^	37.17 ^a^	32.68 ^b^	36.74 ^a^	0.36	<0.05
IGF-1	24.12	23.93	25.16	25.45	25.97	24.82	25.34	0.33	0.66

Note: ^a,b^ Different superscript letters indicate significant differences among different treatment groups (*p* < 0.05). CON treatment group: basal diet; L1 treatment group: 25 mg/kg β-carotene added to the basal diet; L2 treatment group: 4 mg/kg SOD added to the basal diet; L3 treatment group: a mixture of 25 mg/kg β-carotene and 4 mg/kg SOD added to the basal diet; S1 treatment group: 100 mg/kg β-carotene added to the basal diet; S2 treatment group: 14 mg/kg SOD added to the basal diet; S3 treatment group: a mixture of 100 mg/kg β-carotene and 14 mg/kg SOD added to the basal diet; L1–L3: the antioxidant supplementation period began with weaning in the previous breeding cycle and ended with re-breeding after weaning in the present breeding cycle; S1–S3: the antioxidant supplementation period was 7 days before and after weaning in the previous breeding cycle and 7 days before and after farrowing in the present breeding cycle. SEM = pooled standard error of the mean. n = 6.

**Table 11:** Effects of compound antioxidant supplementation on stress state and intestinal barrier function of sows and piglets.

Item	CON	L1	L2	L3	S1	S2	S3	SEM	*p* Value
Serum of sows at 40 days of gestation									
Cortisol, μg/L	313.00 ^a^	304.29 ^a^	305.20 ^a^	267.10 ^b^	308.71 ^a^	322.33 ^a^	308.13 ^a^	3.83	<0.05
LPS, ng/L	694.96 ^a^	653.01 ^ab^	646.31 ^ab^	584.58 ^b^	696.57 ^a^	688.77 ^a^	687.97 ^a^	8.66	<0.05
Serum of sows at farrowing									
Cortisol, μg/L	285.73 ^ab^	228.76 ^c^	243.07 ^bc^	199.25 ^c^	307.76 ^a^	286.86 ^ab^	229.02 ^c^	6.82	<0.05
LPS, ng/L	583.62	554.54	559.63	562.48	607.95	570.79	537.62	8.33	0.40
Placenta									
LPS, ng/L	529.67 ^a^	481.54 ^c^	479.05 ^c^	474.94 ^c^	509.78 ^ab^	513.67 ^ab^	492.36 ^bc^	3.69	<0.05
Serum of piglets at d 14									
LPS, ng/L	404.85	405.77	391.84	395.91	417.74	401.74	390.58	9.36	0.99
Piglet serum at weaning									
LPS, ng/L	505.03 ^a^	430.57 ^ab^	425.07 ^ab^	342.25 ^b^	437.10 ^ab^	431.84 ^ab^	416.58 ^ab^	14.19	<0.05

Note: ^a–c^ Different superscript letters indicate significant differences among different treatment groups (*p* < 0.05). CON treatment group: basal diet; L1 treatment group: 25 mg/kg β-carotene added to the basal diet; L2 treatment group: 4 mg/kg SOD added to the basal diet; L3 treatment group: a mixture of 25 mg/kg β-carotene and 4 mg/kg SOD added to the basal diet; S1 treatment group: 100 mg/kg β-carotene added to the basal diet; S2 treatment group: 14 mg/kg SOD added to the basal diet; S3 treatment group: a mixture of 100 mg/kg β-carotene and 14 mg/kg SOD added to the basal diet; L1–L3: the antioxidant supplementation period began with weaning in the previous breeding cycle and ended with re-breeding after weaning in the present breeding cycle; S1–S3: the antioxidant supplementation period was 7 days before and after weaning in the previous breeding cycle and 7 days before and after farrowing in the present breeding cycle. SEM = pooled standard error of the mean. n = 6.

## Data Availability

The datasets used and/or analyzed during the current study are available from the corresponding author upon reasonable request.

## Supplementary Materials

The following supporting information can be downloaded at: https://www.mdpi.com/article/10.3390/antiox14030359/s1, Figure S1: Correlation analysis between T-AOC and LPS contents in placenta tissue and litter weight gain of offspring; Table S1: Effects of antioxidants on feed intake in sows.
