# Supplementary material for: Transfer of Antioxidant Capacity Through Placenta and Colostrum: β-Carotene and Superoxide Dismutase Collaboratively Enhance Integrated Breeding of Sows and Piglets
Source: Antioxidants (Basel). 2025 Mar 18;14(3):359. doi: 10.3390/antiox14030359 (PMC11939707; doi:10.3390/antiox14030359)
Supplement: Supplementary file 1 [file antioxidants-14-00359-s001.zip › antioxidants-3524590-supplementary.pdf]

Table S1 Effects of antioxidants on feed intake in sows[1].

Item	CON	L1	L2	L3	S1	S2	S3	SEM	*P* value
No. of gilts per group	19	20	18	19	20	18	19		
Average daily feed intake during gestation, kg	2.48	2.48	2.5	2.52	2.51	2.51	2.51	0.006	0.44
Average daily feed intake during lactation, kg	5.23	5.29	5.30	5.53	5.23	5.26	5.46	0.034	0.12
Parity	3.90	4.00	4.00	3.90	4.10	4.00	3.90	0.071	0.99
No. of piglets born in the previous parity	15.05	15.10	15.00	15.11	15.00	15.06	15.05	0.04	0.99

Note: CON treatment group: basal diet; L1 treatment group: add 25 mg/kg β-carotene to the basal diet; L2 treatment group: add 4 mg/kg SOD to the basal diet; L3 treatment group: add a mixture of 25 mg/kg β-carotene and 4 mg/kg SOD to the basal diet; S1 treatment group: add 100 mg/kg β-carotene to the basal diet; S2 treatment group: add 14 mg/kg SOD to the basal diet; S3 treatment group: add a mixture of 100 mg/kg β-carotene and 14 mg/kg SOD to the basal diet; L1-L3: the antioxidant supplementation period begins with weaning in the previous breeding cycle and ends with re-breeding after weaning in the present breeding cycle; S1-S3: the antioxidant supplementation period was 7 days before and after weaning in the previous breeding cycle and 7 days before and after farrowing in the present breeding cycle. SEM = Pooled Standard Error of the Mean. n = 18-20.

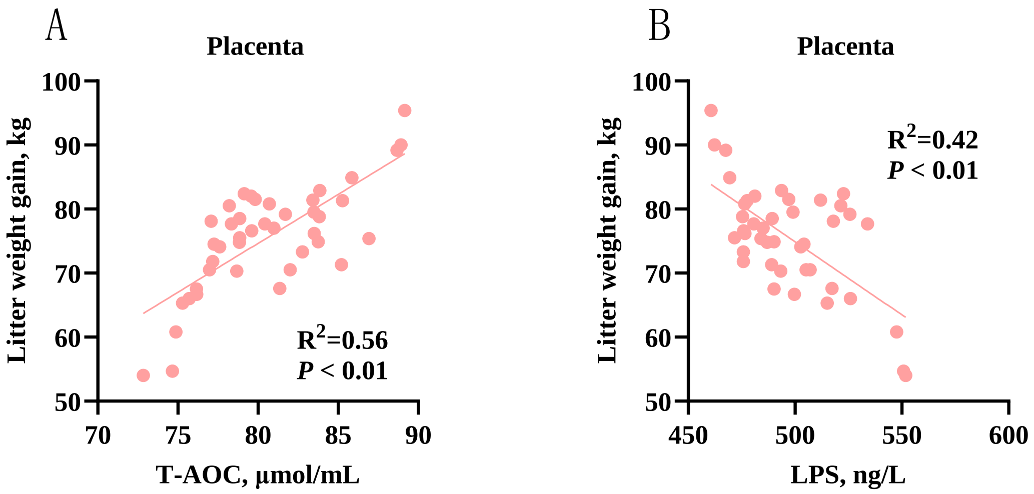

Figure S1. Correlation analysis between T-AOC and LPS contents in placenta tissue and litter weight gain of offspring. (A) Regression analysis between placental T-AOC levels and litter weight gain in piglets. (B) Regression analysis between LPS levels in placenta and litter weight gain in piglets.
